# An Integration of Genome-Wide Association Study and Gene Expression Profiling to Prioritize the Discovery of Novel Susceptibility Loci for Osteoporosis-Related Traits

**DOI:** 10.1371/journal.pgen.1000977

**Published:** 2010-06-10

**Authors:** Yi-Hsiang Hsu, M. Carola Zillikens, Scott G. Wilson, Charles R. Farber, Serkalem Demissie, Nicole Soranzo, Estelle N. Bianchi, Elin Grundberg, Liming Liang, J. Brent Richards, Karol Estrada, Yanhua Zhou, Atila van Nas, Miriam F. Moffatt, Guangju Zhai, Albert Hofman, Joyce B. van Meurs, Huibert A. P. Pols, Roger I. Price, Olle Nilsson, Tomi Pastinen, L. Adrienne Cupples, Aldons J. Lusis, Eric E. Schadt, Serge Ferrari, André G. Uitterlinden, Fernando Rivadeneira, Timothy D. Spector, David Karasik, Douglas P. Kiel

**Affiliations:** 1 Hebrew SeniorLife Institute for Aging Research and Harvard Medical School, Boston, Massachusetts, United States of America; 2 Molecular and Integrative Physiological Sciences Program, Harvard School of Public Health, Boston, Massachusetts, United States of America; 3 Framingham Heart Study, Framingham, Massachusetts, United States of America; 4 Department of Internal Medicine, Erasmus Medical Center, Rotterdam, The Netherlands; 5 Netherlands Genomics Initiative, The Hague, The Netherlands; 6 Department of Twin Research and Genetic Epidemiology, King's College London, London, United Kingdom; 7 Departments of Endocrinology, Diabetes, and Medical Technology and Physics, Sir Charles Gairdner Hospital, Perth, Australia; 8 School of Medicine and Pharmacology, University of Western Australia, Crawley, Australia; 9 Department of Medicine, Division of Cardiovascular Medicine and Center for Public Health Genomics, University of Virginia, Charlottesville, Virginia, United States of America; 10 Department of Biostatistics, School of Public Health, Boston University, Boston, Massachusetts, United States of America; 11 Wellcome Trust Sanger Institute, Hinxton, Cambridge, United Kingdom; 12 Service of Bone Diseases, Department of Rehabilitation and Geriatrics, University Geneva Hospital, Geneva, Switzerland; 13 Department of Human Genetics, McGill University, Montreal, Canada; 14 McGill University and Genome Quebec Innovation Center, Montreal, Canada; 15 Department of Epidemiology, Department of Biostatistics, Harvard School of Public Health, Boston, Massachusetts, United States of America; 16 Departments of Medicine, Human Genetics, and Epidemiology and Biostatistics, Jewish General Hospital, McGill University, Montreal, Canada; 17 Department of Human Genetics, David Geffen School of Medicine, University of California Los Angeles, Los Angeles, California, United States of America; 18 National Heart and Lung Institute, Imperial College London, London, United Kingdom; 19 Department of Epidemiology, Erasmus Medical Center, Rotterdam, The Netherlands; 20 Department of Surgical Sciences, Uppsala University, Uppsala, Sweden; 21 Departments of Medicine, Human Genetics, Microbiology, Immunology, and Molecular Genetics, David Geffen School of Medicine, Molecular Biology Institute, University of California Los Angeles, Los Angeles, California, United States of America; 22 Rosetta Inpharmatics/Merck, Seattle, Washington, United States of America; Queensland Institute of Medical Research, Australia

## Abstract

Osteoporosis is a complex disorder and commonly leads to fractures in elderly persons. Genome-wide association studies (GWAS) have become an unbiased approach to identify variations in the genome that potentially affect health. However, the genetic variants identified so far only explain a small proportion of the heritability for complex traits. Due to the modest genetic effect size and inadequate power, true association signals may not be revealed based on a stringent genome-wide significance threshold. Here, we take advantage of SNP and transcript arrays and integrate GWAS and expression signature profiling relevant to the skeletal system in cellular and animal models to prioritize the discovery of novel candidate genes for osteoporosis-related traits, including bone mineral density (BMD) at the lumbar spine (LS) and femoral neck (FN), as well as geometric indices of the hip (femoral neck-shaft angle, NSA; femoral neck length, NL; and narrow-neck width, NW). A two-stage meta-analysis of GWAS from 7,633 Caucasian women and 3,657 men, revealed three novel loci associated with osteoporosis-related traits, including chromosome 1p13.2 (*RAP1A*, p = 3.6×10^−8^), 2q11.2 (*TBC1D8*), and 18q11.2 (*OSBPL1A*), and confirmed a previously reported region near *TNFRSF11B/OPG* gene. We also prioritized 16 suggestive genome-wide significant candidate genes based on their potential involvement in skeletal metabolism. Among them, 3 candidate genes were associated with BMD in women. Notably, 2 out of these 3 genes (*GPR177*, p = 2.6×10^−13^; *SOX6*, p = 6.4×10^−10^) associated with BMD in women have been successfully replicated in a large-scale meta-analysis of BMD, but none of the non-prioritized candidates (associated with BMD) did. Our results support the concept of our prioritization strategy. In the absence of direct biological support for identified genes, we highlighted the efficiency of subsequent functional characterization using publicly available expression profiling relevant to the skeletal system in cellular or whole animal models to prioritize candidate genes for further functional validation.

## Author Summary

BMD and hip geometry are two major predictors of osteoporotic fractures, the most severe consequence of osteoporosis in elderly persons. We performed sex-specific genome-wide association studies (GWAS) for BMD at the lumbar spine and femor neck skeletal sites as well as hip geometric indices (NSA, NL, and NW) in the Framingham Osteoporosis Study and then replicated the top findings in two independent studies. Three novel loci were significant: in women, including chromosome 1p13.2 (*RAP1A*) for NW; in men, 2q11.2 (*TBC1D8*) for NSA and 18q11.2 (*OSBPL1A*) for NW. We confirmed a previously reported region on 8q24.12 (*TNFRSF11B/OPG*) for lumbar spine BMD in women. In addition, we integrated GWAS signals with eQTL in several tissues and publicly available expression signature profiling in cellular and whole-animal models, and prioritized 16 candidate genes/loci based on their potential involvement in skeletal metabolism. Among three prioritized loci (*GPR177*, *SOX6*, and *CASR* genes) associated with BMD in women, *GPR177* and *SOX6* have been successfully replicated later in a large-scale meta-analysis, but none of the non-prioritized candidates (associated with BMD) did. Our results support the concept of using expression profiling to support the candidacy of suggestive GWAS signals that may contain important genes of interest.

## Introduction

The feasibility of carrying out genome-wide association studies (GWAS) has led to the rapid progression of the field of complex-disease genetics over the past few years. Although the GWAS approach has been successful in identifying novel candidate genes leading to new discovery of pathways that are involved in the pathophysiology of diseases, the genetic variants identified so far only explain a small proportion of the heritability for complex traits [1]. Due to the modest genetic effect size and inadequate power to overcome the heterogeneity of genetic effects in meta-analysis, true association signals may not be revealed based on a stringent genome-wide significance threshold alone [2]. In addition, the majority of the GWAS have not provided much information beyond statistical signals to understand the genetic architecture for those usually novel genes that have not been studied for a particular trait/disease before. Thus, the necessity of incorporating additional information when studying the GWAS has become apparent. Expression profiling with gene signatures of cellular models have been used to characterize gene's involvement in bone metabolism and disease processes. One such approach is parathyroid hormone (PTH) stimulated osteoclastogenesis and osteoblast maturation for osteoblastogenesis [3]. PTH indirectly stimulated osteoclastogenesis via its receptors on osteoblasts, which then signal to osteoclast precursors to stimulate osteoclastogenesis. Impaired osteoblastic differentiation reduces bone formation and causes severe osteoporosis in animals [4]. The *TNFRSF11B/OPG* gene, a well-known candidate gene for osteoporosis, is involved in osteoclastogenesis through the regulation of PTH [5]. Compared to GWAS-identified candidate genes that do not show differential expression in these cellular models, genes like *TNFRSF11B/OPG* with differential expression are more likely to be involved in skeletal metabolism and thus more likely to be truly associated with osteoporosis. Given that the majority of the reported genome-wide significant SNPs are in the intergenic or noncoding regions [6], it is not clear which SNP/gene might be implicated as a causal SNP/gene. Since intergenic or noncoding SNPs do not appear to affect protein sequence, it is likely that these SNPs either are in linkage disequilibrium with the causal variants or located within the transcription regulation elements of nearby genes. The relative quantification of gene transcripts may act as intermediate phenotypes between genetic loci and the clinical phenotypes. Expression quantitative trait loci (eQTL) analysis in specific tissues is a valuable tool to identify potentially causal SNPs [7]–[10]. By integration of genetic variants, transcriptome, and phenotypic data, investigators have the potential to provide much-needed support to prioritize the candidate susceptibility genes identified from GWAS for further validation [11]–[13].

Previously, we conducted a pilot GWAS for osteoporosis-related phenotypes in a small subset of the Framingham study participants [14]. Osteoporosis is a skeletal disorder characterized by compromised bone strength predisposing to an increased risk of fracture. The heterogeneity of osteoporosis has both an environmental and genetic basis. Although bone mineral density (BMD) is frequently used in the diagnosis and prognosis of osteoporosis [15], a growing body of evidence indicates that femoral geometry also contributes importantly to hip fracture risk [16], [17]. Both BMD and hip geometry are strongly heritable, with heritability estimates between 50% and 85% [18]. In an attempt to identify genes that are involved in the regulation of bone health related phenotypes, genetic linkage analyses [19], [20], candidate gene association studies [21] and recent GWAS [22]–[27] have been used to implicate several loci and candidate genes, such as *OPG*/*RANK*/*RANKL* [22]–[24], [28], *LRP5* [22], [23], [29], *LRP4* [23], *ESR1* [23], [30], *VDR* [31], and *SP7* [24], [25]. However, the majority of genes that contribute to genetic susceptibility to osteoporosis remain to be elucidated.

**Figure 1:**
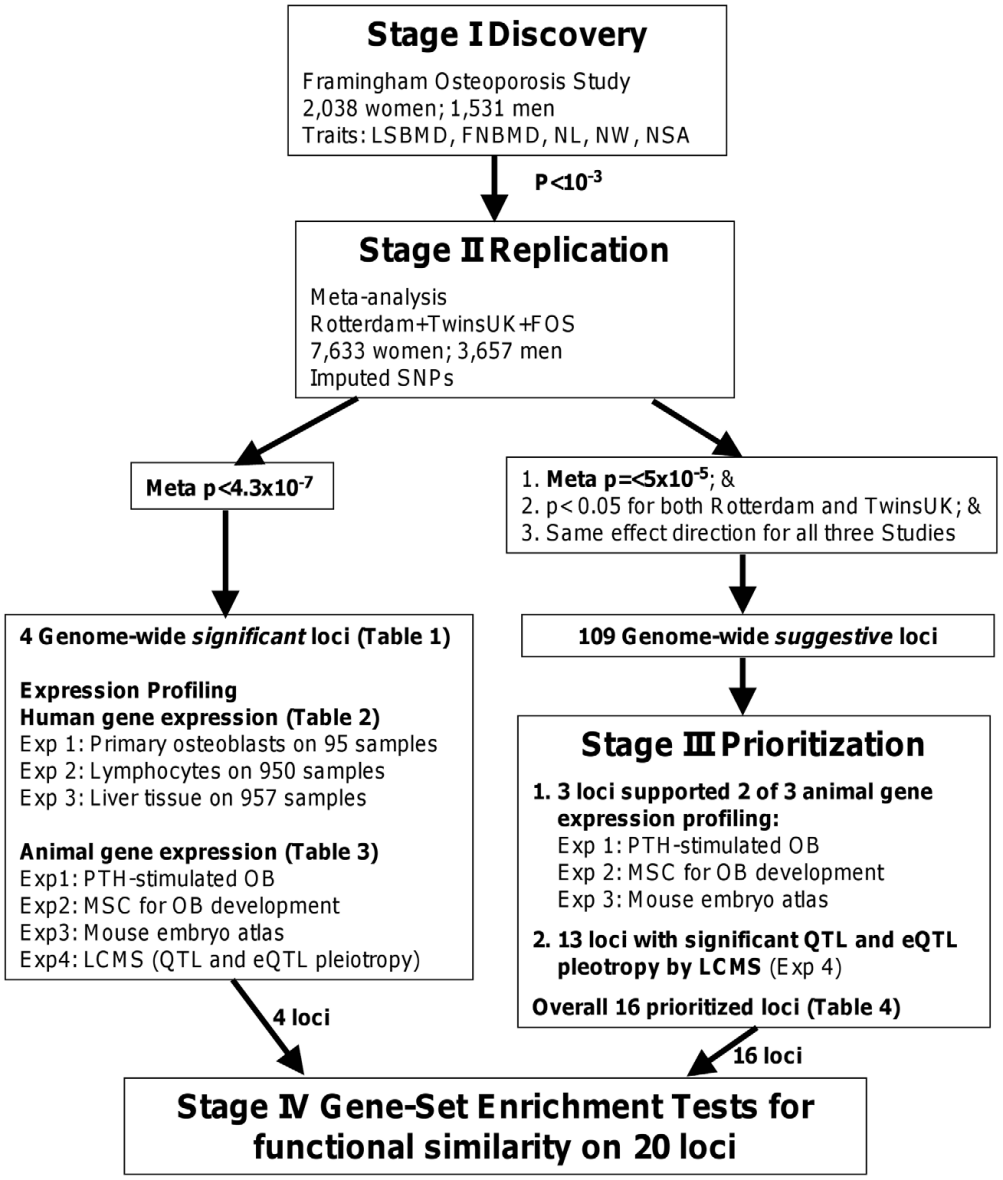
Study design. A four-stage approach was applied. We first performed genome-wide association analyses of the BMD and hip geometry traits in the Framingham Osteoporosis Study as a discovery stage (I) and replicated the top findings by meta-analysis (II), with a subsequent assessment of the functional relevance of the replicated findings (III and IV).

Seeking to extend these initial observations, in the current study, we first performed a large-scale GWAS analysis for BMD and hip geometry in 2,038 women and 1,531 men from the Framingham Osteoporosis Study using 550,000 SNPs, and then replicated the top findings in 5,595 women and 2,126 men from two independent cohorts of Caucasian individuals. We then prioritized the genome-wide association findings by utilizing publicly available experiments relevant to the skeletal system in cellular or whole animal models, and provided supportive biological information for future functional validation of their involvement in bone metabolism. The expression experiments included (1) gene signatures of a mouse embryo expression atlas and mouse cellular models of osteoblastogenesis and PTH- stimulated osteoblasts; (2) eQTL analysis in human primary osteoblasts, lymphocytes and liver tissues; and (3) likelihood-based causality model selection (LCMS) by integrating genetic variants, gene expression profiling, and skeletal phenotypes in inbred mice to identify candidate genes causally related to bone phenotypes. An overview of the study design is provided in Figure 1.

## Results

### Stage I: GWAS in Framingham Osteoporosis Study

**Figure 2:**
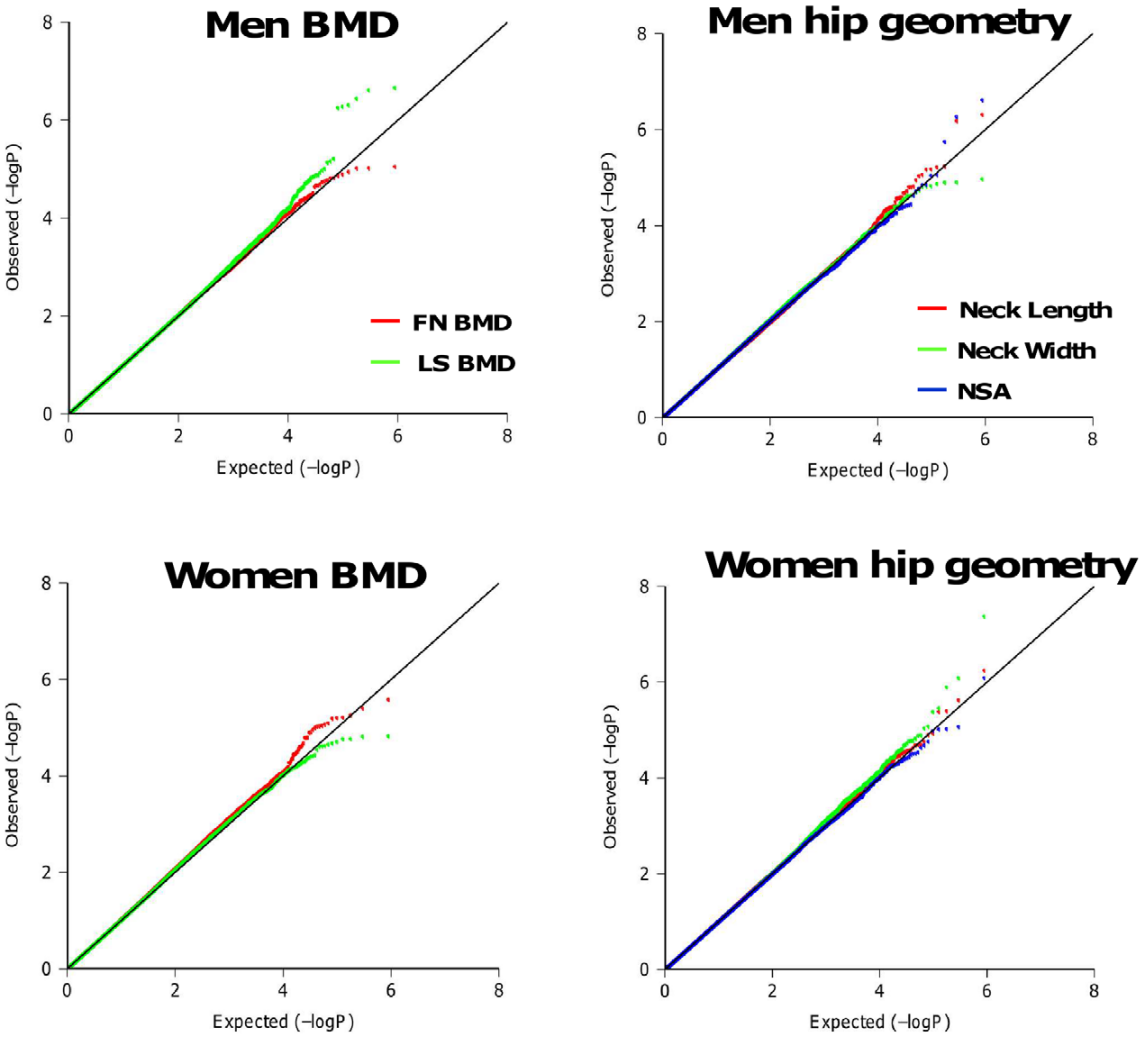
Quantile-Quantile plots for BMD and HSA in additive genetic models. The distributions of observed p-values did not deviate from the null distribution, which rules out systematic bias due to bad genotyping or population substructure in our study samples.

Significant differences of BMD and geometry indices were found between men and women in the Framingham Study with p-values <0.001 (Table S1). Quantile-quantile plots of observed p-values for single SNP association tests under additive genetic effect models are shown in Figure 2. Except for the tail (likely comprising true associations), the distributions of observed p-values did not deviate from the null distribution, which rules out systematic bias due to bad genotyping or population substructure in our study samples. The estimated genome control λ_GC_ for each phenotype ranged from 0.99 to 1.02. The regression coefficients analyzed with and without adjusting for the PCs are highly correlated (r = 0.95–0.98). Thus, we do not expect these principal components to influence our results substantially. SNPs associated with each phenotype at p-values <10^−6^ are listed in Table S2. For women, the most significant association was found with neck width (NW) for SNP rs16965654 (MAF = 0.01) located 13Kb away from the 5′ upstream region of the *WD repeat and SOCS box-containing 1* (*WSB1*) gene on chromosome 17q11.1 (p = 4.15×10^−8^). For men, the most significant association was found with neck-shaft angle (NSA) for SNP rs11573709 (MAF = 0.23) located in intron 7 of the *RAD23 homolog B* (*RAD23B*) gene on 9q31.2 (p = 2.37×10^−7^). We also performed association tests by combining men and women together. The most significant association was found with NW for SNP rs16965654 (p = 6.89×10^−10^).

### Stage II: Meta-Analysis

All genotyped SNPs (n = 431–593 for sex-specific phenotypes) with association test p-values <10^−3^ in Stage I were examined for replication in the Rotterdam Study (both men and women) and TwinsUK Study (women only). We performed meta-analyses by combining results from the Framingham Study and Rotterdam Study in men and all three cohorts in women. P-values <4.3×10^−7^ from meta-analyses are considered as genome-wide significant associations (See statistical methods section for details). We listed the most significant SNP on each chromosome locus with meta-analysis p-values <10^−6^ in Table 1. The most significant association for men was found with NSA for SNP rs2278729 located in the intron 4 of *TBC1D8* on chromosome 2q11.2 (p = 1.48×10^−7^). SNP rs7227401 located in intron 4 of *OSBPL1A* (18q11.2) was found to be strongly associated with NW (p = 4.22×10^−7^) in men. The most significant association for women from meta-analysis was found with LS BMD for SNP rs2062375 located in the intergenic region of *TNFRSF11B* and *COLEC10* genes on chromosome 8q24.12 (p = 2.68×10^−11^). SNP rs494453 located in the intron 2 of *RAP1A* on chromosome 1p13.2 was also strongly associated with NW (p = 2.80×10^−7^). The association became more significant for SNP rs494453 when combining women and men together (p = 3.6×10^−8^). None of the above associated SNPs are exonic coding SNPs. For SNPs listed in Table 1, no significant heterogeneity across studies was found and the p-values (as well as regression coefficients) were not changed with or without adjustment of body weight. The quality scores of imputed SNPs in Table 1 were >0.98 (IMPUTE confidence score) for the TwinsUK Study and >0.84 (MACH variance ratio) for the Rotterdam Study.

**Table 1:** The most significant SNP in each locus with joint-analysis p-value <10^−6^.

*SNP*	*Allele*	*Cyto-genetic Loci*	*Position*	*Gene^a^*	*Location^b^/Distance (Kbps) to nearby gene*	*Trait^c^*	*Framingham*			*Replication P-valuesd,e*			*Meta-analysis^f^*	
							*MAF*	*Beta*	*P-value*	*Rotterdam*	*TwinsUK*	*Meta P-value*	*Effect direction*	*P-value*
***Men***														
rs2278729	G -> A	2q11.2	101035289	TBC1D8	Intron 4	NSA	0.33	-0.19	3.07E-06	5.19E-03^e^	n.a.	5.19E-03	- -	**1.48E-07**
rs12808199	A -> G	11p12	39284535	LRRC4C*	987.7	FN BMD	0.43	-0.21	2.06E-05	3.88E-03	n.a.	3.88E-03	- -	**8.89E-07**
rs7227401	G -> T	18q11.2	20192656	OSBPL1A	Intron 4	NW	0.39	0.17	3.58E-06	8.57E-03^e^	n.a.	8.57E-03	+ +	**4.22E-07**
***Women***														
rs494453	T -> C	1p13.2	111993645	RAP1A	Intron 2	NW	0.24	0.14	2.19E-04	3.28E-04^e^	2.59E-01^e^	2.77E-04	+ + +	**2.80E-07**
rs12151790	G -> A	2q37.1	234875466	SPP2*	251.4	FN BMD	0.04	0.40	1.88E-06	1.60E-02^e^	4.52E-01^e^	2.58E-02	+ + +	**4.64E-07**
rs2062375	C -> G	8q24.12	120046973	TNFRSF11B*	13.4	LS BMD	0.45	0.14	8.07E-06	1.59E-03^e^	4.58E-05^e^	2.47E-07	+ + +	**2.68E-11**
rs17184557	T -> A	18q22.2	65293837	DOK6	Intron 1	LS BMD	0.23	0.13	9.19E-04	1.62E-02^e^	3.13E-03^e^	1.45E-04	+ + +	**8.81E-07**

**a** SNP locates within a gene.

*: For most significantly associated SNP located on the intergenic regions, the closest nearby gene was listed.

**b** The SNP location is shown if SNP locates within a gene. The distance (Kb) from an intergenic SNP to the closest gene is shown if SNP locates in the intergenic regions.

**c** NL: Neck Length; NW: Neck Width; NSA: Neck Shaft Angle.

**d** In men, data were only available from Framingham and Rotterdam studies. Meta-analysis p-values of the replication in men are the p-values from Rotterdam Study.

**e** Imputed SNPs: IMPUTE confidence score > 0.98 for TwinsUK; MACH variance ratio > 0.84 for Rotterdam Study.

**f** Effect direction: In the order of Framingham, Rotterdam and TwinsUK studies.

### eQTL in Multiple Human Tissues

*Cis*-eQTLs were analyzed for eight candidate genes located within 500 kb in four genome-wide significant loci (Table 2). All eight candidate genes were expressed in bone tissue estimated by either expressed sequence tag (EST) in the CGAP EST cDNA library (Figure S1) or human primary osteoblast samples (Table 2). However, since transcripts were not presented on expression arrays, expression of *TBC1D8* was not available in human primary osteoblast samples. P-values <0.005 estimated by false discovery rate (FDR) were considered as significant. SNP rs494453 was found to be significantly associated with transcript levels of the *RAP1A* gene. Allele C of rs494453 is in LD with allele A of rs3767595 (haplotype). The haplotype CA was associated with lower expression of *RAP1A*, but higher NW (stronger bone structure) in women. We also performed eQTL analyses in human lymphocytes and liver tissue. Expression level of the *RAP1A* gene was not available for either lymphocytes or liver tissue. SNPs on chromosome 2q11.2 (*TBC1D8* and *RLP31*) and 8q24.12 (*TNFRSF11B*) loci were associated with gene expressions in lymphocytes (Table 2). The most significant eSNP was found for SNP rs2278729 (chromosome 2q11.2) with *TBC1D8* expression in lymphocytes (p = 2.58×10^−10^) and liver tissue (p<10^−16,^ Figure S3). Allele A of rs2278729 was associated with smaller NSA in men and also with lower expression of *TBC1D8* transcript. The same allele A was also associated with lower *RPL31* expression in lymphocytes and was marginally significant in osteoblasts. Consistency between the direction of effect on transcript levels in lymphocytes and LS BMD was observed for *TNFRSF11B* at the chromosome 8q24.12 locus, which confirmed a previous report that increased *TNFRSF11B* expression levels have been shown to inhibit bone resorption [32]. A previous study also demonstrated that alleles associated with decreased BMD were associated with differential allelic expression of the *TNFRSF11B* in lymphocytes [22]. However, we did not observe associations of genome-wide significant SNPs in/near the *TNFRSF11B* gene region with *TNFRSF11B* expression levels in human primary osteoblasts, possible due to lack of power.

**Table 2:** *Cis*-expression quantitative trait locus analyses of genome-wide significant SNPs (p < 4.3 x 10^-7^) selected from Table 1 with transcript levels in human lymphocytes and primary osteoblasts.

*Target SNP*	*Allele, Effect direction*	*Gene^a^*	*Nearby Transcripts*	*Distance to transcript (Kb)*	*Lymphocytes^b^*		*Primary osteoblasts^c^*			
					*P-value*	*Effect direction*	*Surrogate SNPs*	*r^2^, Distance (Kb) to transcript*	*P-value*	*Allele^d^, Effect direction*
rs2278729	G -> A, -	TBC1D8	TBC1D8	**Intron 4**	**2.58E-10**	-	rs6543018	0.75, Intron 1	n.a.	n.a.
			RPL31	**32.7**	**1.11E-16**	-	rs6543018	0.75, 92.7	4.06E-02	T -> C, -
rs7227401	G -> T, +	OSBPL1A	OSBPL1A	Intron 4	n.s.	n.s.	rs7226913	1.00, Intron 4	5.69E-01	C -> T, +
			IMPACT	68.0	8.70E-03	-	rs7226913	1.00, 68.3	6.71E-01	C -> T, +
rs494453	T -> C, +	RAP1A	RAP1A	Intron 2	n.a.	n.a.	**rs3767595**	**0.61, Intron 2**	**3.98E-03**	**G -> A, -**
			ADORA3	150.1	n.s.	n.s.	rs10489469	0.51, 32.3	3.11E-02	G -> T, +
rs2062375	C -> G, +	TNFRSF11B	TNFRSF11B	**13.4**	**6.53E-06**	+	rs1032128	0.84, 21.9	2.47E-01	A -> G, -
			COLEC10	101.7	n.s	n.s.	rs6469804	0.91, 5.2	8.20E-01	A -> G, -

**a** TNFRSF11B: The most significantly associated SNP located on the intergenic regions, the closest nearby gene was selected.

**b** Dataset with available imputed SNPs.

**c** Dataset without available imputed SNPs. Surrogate SNPs for the target SNP was used. r2 was estimated between target SNP and surrogate SNP.

**b,c** Experiments were performed in different study populations.

**d** The first allele is in LD with the major allele of the target SNP (haplotype). For example: Allele G of SNP rs2278729 is in LD with the allele T of rs6543018.

**n.s.** P-value > 0.005 (FDR).

**n.a.** Expression level was not available, since transcripts were not present on expression arrays.

### Mouse Expression Profiling Experiments

We investigated the candidate genes corresponding to the genome-wide significant SNPs in 4 chromosomal regions by looking at reported gene functions (including biological processes, canonical pathways and organism processes in human and mouse), microRNA targets and gene-related human diseases (Table S3). Except for the *TNFRSF11B* gene, there were few additional data regarding the potential biological significance of other candidate genes being involved in skeletal development and bone remodeling; therefore, we performed additional analyses on expression profiles in animal experiments (Table 3). In experiment 1, we found that PTH negatively regulated expressions of *OSBPL1A* and *TNFRSF11B*. *RPL31*, *IMPACT and RAP1A* genes were expressed in PTH stimulated osteoblasts, but not regulated by PTH. *TBC1D8* were not expressed in PTH stimulated osteoblasts. In experiment 2, we analyzed the differential expression of candidates during osteoblast maturation. As a quality control measure, we looked at a number of known osteoblast markers, including runt-related transcription factor 2 (*Runx2*), collagen type 1, alpha 1 (*Col1a1*), collagen type 1, alpha 2 (*Col1a2*), osteocalcin, osteopontin and osteonectin. The expected expression patterns (differential expression during maturation) were observed in all cases. We observed that the expression of *OSBPL1A*, *IMPACT* and *COLEC10* was significantly different across a time course (Day 4, 5, 6, 8, 16, 25 and 30 post-differentiation) of osteoblast development (p<0.0083). In the third experiment using the LCMS algorithm in the B6XC3H F2 intercross mice, we found that *OSBPL1A*, *IMPACT*, *RAP1A* and *COLEC10* genes were predicted to be causally linked with bone phenotypes (detailed phenotypes listed in Table S4) based on the evidence of significantly pleiotropic effects on trait QTL and eQTL.

**Table 3:** Expression profiles for 4 genome-wide significant loci in mice osteoblast gene expression experiments and Likelihood-based Causality Model Selection (LCMS) regulatory network analysis in inbred mice.

*Cytogenetic Loci*	*The closest Gene^a^*	*Nearby Transcripts^b^*	*Experiment 1^c^*	*Experiment 2^d^*	*Experiment 3^e^*	
			*PTH stimulated primary osteoblasts*	*Differential expression during osteoblast development*	*LCMS analysis for significant eQTL and trait QTL pairs*	
			*Expression, Direction*	*P-value*	*# Traits*	*Max # pairs*
2q11.2	TBC1D8	TBC1D8	0	8.23E-01	0	0
		RPL31	++	n.a.	n.a.	n.a.
18q11.2	OSBPL1A	OSBPL1A	+++ ↓	**4.59E-03**	**1**	**2**
		IMPACT	++	**3.17E-03**	**1**	**2**
1p13.2	RAP1A	RAP1A	++	2.12E-02	**3**	**3**
		ADORA3	n.a.	n.a.	0	0
8q24.12	TNFRSF11B*	TNFRSF11B	+++ ↓	7.61E-01	0	0
	COLEC10*	COLEC10	n.a.	**1.54E-03**	**1**	**2**

**a** SNP locates within a gene.

*: For most significantly associated SNP located on the intergenic regions, the closest nearby gene was listed.

**b** Transcripts: Transcripts from (1) the closest gene; or (2) genes with target SNP located less than 500K bps on the 5' upstream flanking region.

**c** PTH stimulated primary osteoblasts: 0: not expressed; ++: expression level > 100 in all 3 replicates; +++: expressed in all 3 replicates and regulated by PTH.

**d** ANOVA was used to test the differential expression across 7 time points (Day 4, 5, 6, 8, 16, 25 and 30 post-induction) during osteoblast development. Bold: p-value < 0.0083 ( = 0.05/6 available transcripts).

**e** LCMS analysis: Likelihood-based Causality Model Selection to predict candidate genes causally linked with bone phenotypes. Six bone related traits were tested. For each trait test, at least 2 significant pleiotropy of eQTL and trait QTL pairs was considered evidence for a causally relation to the candidate gene.

**c,d,e** Results of experiment 1,2 and 3 were obtained from different mice strains and different laboratories.

**n.a.** Expression level is not available, since transcript is not presented on expression arrays.

### Prioritization of the Genome-Wide Suggestive Candidate Genes

A total of 109 suggestive genome-wide associated regions/genes (most significant SNP with meta-analysis 4.3×10^−7^< p-value ≤5×10^−5^) were selected based on the criteria that p-values showed nominal association in the Framingham, Rotterdam and TwinsUK studies. Among them, 16 candidate genes were prioritized with results either involving the differential expression in osteoblasts or causally linked (LCMS algorithm) with bone phenotypes in mice (Table 4). Among 16 prioritized candidate genes/loci, *PPAP2B*, *GPR177*, *TGFBI*, *DOCK1*, *SOX6* and *PDGFD* gene expressions were regulated by PTH in osteoblasts. Significant differential expression during osteoblast development was found for *GPR177*, *TGFBI*, *SOX6* and *CDH2* genes. *IRX2*, *TGFBI* and *CDH2* genes showed strong expression in the skeleton compared to 24 other subsets of organ/tissue systems of the mouse embryo. Using the LCMS algorithm in inbred mice, 12 genes were predicted to be causally linked with bone phenotypes (detailed phenotypes listed in Table S4). All of the prioritized candidate genes are expressed in bone tissues. 10 genes were found to be expressed in human bone tissue from the CGAP EST cDNA library (Figure S1) and the remained genes (*HECW2*, *CASR*, *MMRN1*, *IRX2*, *SOX6* and *SALL1*) were found to be expressed in human primary osteoblasts.

**Table 4:** Prioritized suggestive genome-wide loci based on mice experiments of Likelihood-based Causality Model Selection (LCMS) regulatory network analyses, gene expression signature profiles in osteoblasts, and transcriptome atlas of mouse embryos.

*SNP^a^*	*Cytogentic Loci*	*CHR. Position*	*Gene b*	*SNP Location/ Distance to gene (Kb) c*	*Meta-analysis for selected SNPs*					*Animal Expression profile Experiments*						
					*TRAIT d*	*Framingham Cohort*			*Meta Analysis*	*Experiment 1 e*	*Experiment 2 f*	*Experiment 3 g*			*Experiment 4 h*	
						*Minor Allele*	*MAF*	*Effect*	*P-values*	*PTH stimulated primary osteoblasts*	*Differential expression during osteoblast development*	*LCMS regulartory networks for significant eQTL and trait QTL pairs*			*Transcriptome atlas for mouse embryo RNA in-situ hybridization assays*	
										*Expression*	*P-value*	*# Traits*	*pairs*		*Regional Signal*	*Skeletal System*
rs10789021	1pter-p22.1	56676131	PPAP2B *	141.7	NL, men	A	0.34	0.19	4.83E-05	**+++ ↑**	1.78E-02	**1**		**3**	no	no
rs6588313	1p31.3	68543872	GPR177 *	73.0	LSBMD, women	G	0.32	0.16	4.87E-06	**+++ ↓**	**3.80E-04**	n.a.		n.a.	n.a.	n.a.
rs1350102	2q32.3-q33.1	196871047	HECW2	Intron 13	FNBMD, men	G	0.10	-0.23	3.70E-05	n.a.	4.98E-01	**2**		**3**	no	no
rs9866419	3q13	123422899	CASR	Intron 1	LSBMD, women	G	0.30	-0.12	1.09E-05	0	8.99E-01	**2**		**3**	no	no
rs1442138	4q22	91035317	MMRN1	Exon 1 (T-> A)	FNBMD, men	G	0.05	0.27	4.26E-05	n.a.	9.57E-02	**1**		**3**	Strong 2,3, 8	no
rs2455455	5p15.33	2671960	IRX2 *	132.8	LSBMD, men	T	0.41	-0.15	1.50E-05	0	3.53E-01	**1**		**2**	Strong, 1,2,3,6,8	**Axial**
rs6450853	5p13.3	31713996	PDZD2 *	120.8	NL, women	G	0.35	0.13	2.15E-05	n.a.	2.76E-01	**1**		**2**	no	no
rs917303	5q31	135445931	TGFBI *	53.3	NSA, men	A	0.34	0.15	2.43E-05	**+++ ↑**	**1.37E-05**	0		0	Strong 1, 5-8	**Axial**
rs11597670	10p12	18569082	CACNB2	Intron 2	NSA, man	G	0.03	0.35	5.00E-05	0	1.93E-01	**3**		**3**	Moderate 2	no
rs11245204	10q26.13-q26.3	128555066	DOCK1 *	103.9	NSA, men	A	0.45	0.15	4.92E-05	**+++ ↓**	8.55E-01	**2**		**3**	no	no
rs17463551	11p15.3	16387773	SOX6	Intron 1	LSBMD, women	T	0.09	0.21	2.85E-06	**+++ ↓**	**3.18E-04**	0		0	no	no
rs2087324	11q22.3	103711091	PDGFD *	170.9	FNBMD, men	T	0.33	0.13	2.32E-05	**+++ ↓**	7.73E-01	**1**		**2**	no	no
rs10895656	11q22.3	103722691	PDGFD	182.5	LSBMD, men	C	0.42	0.16	2.57E-06	**+++ ↓**	7.73E-01	**1**		**2**	no	no
rs3784131	14q23-q24.2	68032257	RAD51L1	Intron 10	NL, men	G	0.12	-0.22	3.14E-06	0	9.23E-02	**3**		**2**	Moderate 3-5, 8	no
rs17201113	16q12.1	50665312	SALL1*	384.1	NL, men	A	0.01	1.50	5.42E-07	0	1.31E-02	**1**		**2**	Strong, 2,3,6,8	no
rs4843204	16q24.2	85584221	FBXO31 *	390.6	NL, men	C	0.05	-0.34	3.03E-05	**+**	1.47E-02	**2**		**3**	no	no
rs11664087	18q11.2	23862333	CDH2	Intron 2	NSA, women	A	0.02	0.25	2.55E-06	**+**	**4.65E-05**	n.a		n.a	Strong 1,2,4,5,6,8	**Rib**

**a** Suggestive genome-wide significant SNPs: See text for criteria.

**b** SNP locates within a gene.

*: For the most significantly associated SNP located on the intergenic regions, the closest nearby gene was selected.

**c** SNP location is shown if SNP locates within a gene. The distance (Kb) from an intergenic SNP to the closest gene is shown if SNP locates in the intergenic regions.

**d** NL: Neck Length; NW: Neck Width; NSA: Neck Shaft Angle.

**e** PTH stimulated primary osteoblasts: 0: not expressed; +: expression level < 100 in all 3 replicas; ++: expression level > 100 in all 3 replicas;+++: expressed in all 3 replicas and regulated by PTH. ↑: Up-regulated by PTH; ↓ Down-regulated by PTH.

**f** ANOVA was used to test the differential expression across 7 time points (Day 4, 5, 6, 8, 16, 25 and 30 post-induction) during osteoblast development. P-value cut off is equal to 4.59E-04 ( = 0.05/109).

**g** LCMS analysis: Likelihood-based Causality Model Selection. Six bone related traits were tested. For each trait test, at least 2 significant pleiotropy of eQTL and trait QTL was considered evidence for a causally relation to the candidate gene. Detailed results are shown in Table S4.

**h** RNA in situ hybridization from E14.5 wild type murine embryos. Average expression strength calculated from the assay annotation grouped into 25 subsets of organ/tissue systems of the mouse anatomy. In this table, we only display subsets including 1: Skeleton; 2: Brain and CNS; 3: Spinal cord; 4: PNS; 5: Ganglia; 6:Limb; 7: Skeletal muscle; and 8: Others.

**no** gene expression is not detectable. n.a.: Expression level is not available, since transcripts are not presented on expression arrays.

**e,f,g,h** Results of experiments were obtained from different mice strains and different Laboratories.

### Gene Set Enrichment Test

To test the probability of our candidate genes clustering into a particular biological pathway, we performed a gene set enrichment test on 24 candidate genes (20 loci) from Table 2 and Table 4. Due to lack of biological or functional annotation, *IRX2* and *FBXO31* genes were excluded from analyses. We found a significant clustering (Fisher exact test p = 1.65×10^−4^; Benjamini-Hochberg multiple testing corrected p-value = 0.03) of genes involved in adhesion of cells, including *CASR*, *CDH2*, *PPAP2B*, *RAP1A*, *TGFBI* and *TNFRSF11B* genes. We also estimated expression abundance by number of expressed sequence tag (EST) sequences per 200,000 tags in the CGAP EST cDNA library for these 24 candidate genes. Among 48 human tissues and organs, candidate genes were expressed in bone (17 candidate genes), liver (22 candidate genes), muscle (18) and adipose tissue (12) (Figure S1 and Figure S2). Expression levels of *RAP1A* (p = 2.51×10^−4^), *RPL31* (p = 3.03×10^−7^) and *TNFRSF11B* (p = 1.69×10^−3^) genes showed over-representation in bone (Figure S1).

## Discussion

In this study we performed sex-specific genome-wide association studies for BMD at the LS and FN skeletal sites as well as geometric indices of the hip in adults from the Framingham Osteoporosis Study and then replicated the top finding in two independent studies. As a result of meta-analyses on 7,633 women and 3,657 men, we discovered three novel genome-wide significant loci, including chromosome 1p13.2 *RAP1A* locus (p = 3.62×10^−8^; NW in men and women combined), 2q11.2 *TBC1D8* locus (p = 1.48×10^−7^, NSA in men) and 18q11.2 *OSBPL1A* locus (p = 4.22×10^−7^, NW in men). We also confirmed *TNFRSF11B* gene on chromosome 8q24.12 to be associated with LS BMD in women only (p = 2.68×10^−11^).

The *RAP1A* gene (chromosome 1p13.2) was predicted to be causally linked with bone phenotypes in B6xC3H F2 intercross mice. Compared to other tissues, expression levels of *RAP1A* showed over-representation in human bone tissue. An eSNP (rs494453) located in intron 2 of *RAP1A* gene was also found to be significantly associated with *RAP1A* gene expression in human primary osteoblasts. A marginally significant differential expression during osteoblast maturation was also found in our study. RAP1A, a GTPase that mediates calcium signal transduction, has been found to mediate activities of JnK [33]. JnK has been reported to be involved in late stage osteoblast differentiation [34] and apoptosis of osteoblasts [35]. Therefore, variants in the *RAP1A* gene may change the activities of JnK and then impact osteoblast maturation. Further experiments are necessary to explore the role of the *RAP1A* gene. Both *OSBPL1A* and *IMPACT* genes located in chromosome 18q11.2 region were predicted to be causally linked with bone phenotypes in mice. Expressions of both genes were found to be significantly differential during osteoblast maturation. However, only expression of the *OSBPL1A* gene in osteoblasts was regulated by PTH. No significant eQTL was found in this region. Given the genome-wide significant SNPs were located in the *OSBPL1A* gene, we still cannot rule out the involvment of the nearby *IMPACT* gene. In addition, an *in vitro* study has shown that DDIT3 over-expression enhances osteoblastic differentiation in ST-2 stromal cells, a mechanism that may involve the formation of heterodimers with C/EBP-β and the sensitization of the BMP/Smad signaling pathway [36]. IMPACT protein has found to decrease expression of mouse DDIT3 protein [37]; therefore, *IMPACT* may negatively regulate bone formation.

We estimated the statistical power of our meta-analysis at an α-level of 10^−7^. In women, the power was 62–99% and >80% for effect size (h^2^) equal to 1% and 2%, respectively. In men, the statistical power was 35–75% and >70% for effect size equal to 1% and 2%, respectively. Inadequate statistical power seems to be one of the limitations in our study. Therefore, we prioritized 16 candidate genes/loci out of 109 suggestive genome-wide suggestive candidate genes (4.3×10^−7^<p≤5×10^−5^) based on the expression profiling and the LCMS modeling relevant to the skeletal system. Among 16 prioritized candidate genes/loci, *PPAP2B*, *GPR177*, *SOX6* and *CDH2* genes have been reported to be involved in Wnt-signaling. *CASR*, *TGFBI* and *CACNB2* genes are involved in ossification, endochondrial bone formation in cartilage and calcium ion transportation, respectively (Table S5). *CASR* knockout mice have demonstrated decreased bone density and abnormal bone mineralization [38]. Variants in *GPR177*, *SOX6* and *CASR* genes were associated with LSBMD in women. Variants in *GPR177* and *SOX6* (2 out of 3 above genes) have been successfully replicated in a large-scale meta-analysis of BMD on 19,195 Caucasian subjects (majority of whom were women) with association p-values <10^−9^ [27], but none of the non-prioritized candidates (associated with BMD) did. These results support the concept of our prioritization strategy. Candidate gene/SNP prioritization strategies by gene expression and bioinformatic databases leverage the complexity of the disease phenotypes, which offers some advantages over traditional association studies that rely on strictly p-value driven approaches. A recent study demonstrated that using functional information in published references to identify the key biological relationships between genes was able to predict the success of validation in replication genotyping [39], which also provides additional evidence for the soundness of using biological functional relevance to prioritize candidate genes from GWAS for future validation.

We exploited eSNP/eQTL in multiple human tissues. Given that (1) disease-related human tissues are often difficult to obtain for research purposes; (2) eQTL analysis requires a large sample size to reach the statistical power necessary to observe subtle changes in gene expression [40]; and (3) all of the selected candidate genes were expressed in bone tissues, we believe that performing eQTL in multiple tissues, although not replacing eQTL analysis in bone tissue, does provide complementary information. Genetic control of biological functions may be tissue-specific. Analysis of *cis*- eQTL in the tissue type directly relevant to the phenotype has been generally shown to be more informative than the same analysis in unrelated tissue types (such as blood). However, studies have found that *cis*-eQTLs are conserved across tissues, when genes are actively expressed in those tissues [10], [12], [13], [41]–[44]. eQTL analyses in liver, adipose, brain and muscle tissues from the same individual mice suggested that, for a gene exhibiting significant *cis*-eQTL associations in one tissue, 63–88% (dependent on tissue types) of them also exhibit *cis*-eQTL associations in another tissue [42]. Two recent studies, quantifying allele-specific gene expression in four human cell lines (lymphoblastoid cell, two primary fibroblasts and primary keratinocytes) from the same individuals, observed that only 2.3–10% of the mRNA-associated SNPs showed tissue-specific *cis*-expression across these cell lines [43], [44]. They also found that the variation of allelic ratios in gene expression among different cell lines was primarily explained by genetic variations, much more so than by specific tissue types or growth conditions [43]. Among the highly heritable transcripts (within the upper 25th percentile for heritability), 70% of expression transcripts that had a significant *cis*-eQTL in adipose tissue also had a significant *cis*-eQTL in blood cells [45]. Comparing eQTL in human primary fibroblasts, Epstein-Barr virus-immortalized B cells and T cells revealed that cell-type-shared eQTL tend to have larger effects, higher significance and to cluster tightly around the transcription start site [46]. As for bone tissue, comparing gene expression in 58 human primary osteoblast samples and 57 lymphoblastoid cell samples, despite tissues obtained from different individuals, indicated that overall, there is a large overlap in genes expressed in these two cell types, as well as the associated functional pathways [47]. 60% of the top 100 eSNP in human lymphoblastoid cells also showed associations in human primary osteoblasts, which indicated that both tissue-independent and dependent eSNP were observed in primary osteoblasts and lymphoblastoid cells [47]. Taken together this evidence suggests that if genes are expressed across tissues, their allele-specific expression can be preserved and highly correlated across tissues. Thus, the expression of a gene in liver or other non-bone tissues may not directly cause a change in bone; however, it is possible that its allele-specific expression in liver is highly correlated with allele-specific expression in bone. Because of these correlations it is possible that a gene's expression in adipose or liver can serve as surrogate markers to study the eQTL; however, the real causal relationship would be occurring in bone.

It is important to note that a lack of evidence from mining publicly available gene expression experiments does not necessarily exclude a gene's involvment in skeletal metabolism, given that (1) experimental models such as osteoblastogenesis or early skeletal development, do not represent all relevant processes related to osteoporosis; (2) variation in a gene leading to disease may affect protein function but not expression; and (3) absence of association between a transcript and disease-associated SNP may be due to limited statistical power or under different environmental conditions. An inherent limitation in most of the cell line *in-vitro* gene expression profiling experiments, such as our PTH treated osteoblasts or Epstein-Barr virus-immortalized lymphoblastoid cell lines used to perform eQTL analysis in most of the GWAS, is that the expression profiling of the cultured cells may be varying from actual expression within *in-vivo* cells [10]. An additonal challenge of using available experimental data is that most of the studies performed gene profing using commercialized “genome-wide” chips, which usually have a fixed number of genes and often do not include all set of genes of given interest. Therefore, prioritization of candidate genes will be biased towards well-studied genes.

Few published GWAS have addressed the potential sex-difference in genetic risks of diseases. BMD and hip geometry for men and women are known to differ, as does the prevalence of osteoporotic fractures [48]. Gender differences in the heritability of osteoporosis-related phenotypes have been reported (reviewed in [49]). In the current study, few overlapping associated SNPs between men and women were found, which may be expected based on epidemiological and clinical data and may also be due to lack of power. Sex-specific associations may be due to lifestyle and environmental variation between men and women. However, it also indicates that common genetic effects for both genders may be relatively rare and therefore, larger sample sizes of men and women is needed to detect their existence. Another limitation is that we are unable to distinguish the gender-specific differential expressions, since gene expression is measured in a pooled mixture of osteoblasts from males and females, although, differentiated expression between sexes is actually less likely to occur *in*-*vitro*.

In summary, our study identified three novel genome-wide significant loci and prioritized 16 genome-wide suggestive candidate genes for BMD and hip geometry traits. Beyond generating a list of top associated SNPs by statistical signals, we highlighted the efficiency of our approach to reasonably prioritize association findings by utilizing publicly available expression profiling relevant to the skeletal system in cellular or whole animal models; and to provide supportive biological information for future functional validation of their involvements in bone metabolism. Resequencing of these loci is needed to determine the causal variants and genes, along with experimental functional studies to establish their precise mechanism linked to bone health related phenotypes.

## Materials and Methods

A four-stage approach was applied (Figure 1). We first performed genome-wide association analyses of the BMD and hip geometry traits in the Framingham Osteoporosis Study (discovery stage I) and replicated SNPs with association test p<10^−3^ using meta-analysis by combining results from the Rotterdam Study, TwinsUK Study and Framingham Study (Stage II), with a subsequent assessment of the functional relevance of the replicated findings (Stage III and IV). All study subjects were of self-reported Caucasian origin.

### Discovery Stage (I)

#### Framingham Osteoporosis Study

The Framingham Osteoporosis Study (FOS) is an ancillary study of the Framingham Heart Study (FHS) [50]. The current study involved participants from the FHS Original Cohort [51] and Offspring Cohort [52]. The Original Cohort participants underwent bone densitometry by DXA with a Lunar DPX-L (Lunar Corp., Madison, WI, USA) during their examination 22 (1992–1993) and examination 24 (1996–1997). The Offspring Cohort was scanned with the same machine at or between their examination cycle 6 or 7 (between 1996 and 2001). Participants in current study were a subset from the Original and Offspring cohorts who provided blood samples for DNA and had DXA scans of the hip and spine. Other than being selected on the basis of having bone phenotypes and DNA, the participants were not selected on any trait. In total, 2,038 females and 1,531 males had both available genotyping and bone phenotypes (Table S1). Informed consent was obtained from participants before entry into the study. This study was approved by the Institutional Review Boards for Human Subjects Research at Boston University and the Hebrew Rehabilitation Center.

#### Quantitative bone phenotypes and covariates

**Figure 3:**
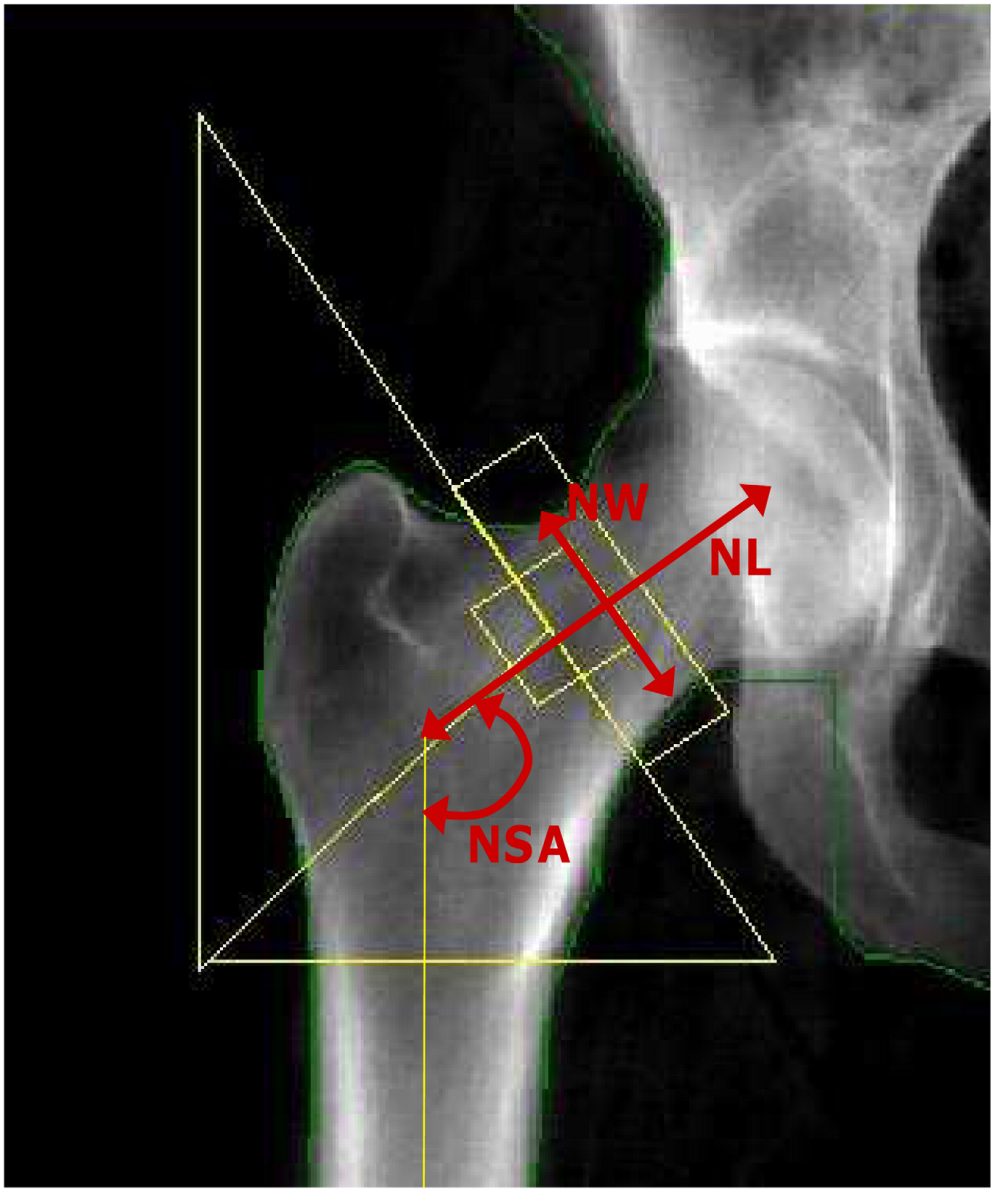
Hip geometry indices. Red arrows indicate three hip geometry indices in a typical DXA image of the right hip. NL: Femoral neck length (cm); NW: Narrow neck width (cm); and NSA: Neck-shaft angle.

Femoral neck (FN) and L2–L4 lumbar spine (LS) BMD (g/cm^2^) was measured by DXA with a Lunar DPX-L for all FOS participants. The coefficients of variation (CV) in normal subjects for the DPX-L have been previously reported to be 0.9% for the LS and 1.7% for the FN BMD [50]. A hip structure analysis computer program (HSA) [53] was used to derive a number of hip geometry variables from the femoral DXA scans. The regions assessed were the narrowest width of the femoral neck (NN), which overlaps or is proximal to the standard Lunar femoral neck region. Although the program derived a number of structural variables, in the current study we only performed analyses for femoral neck length (NL, cm), neck-shaft angle (NSA), as well as subperiosteal diameter (neck width, NW, cm), which are direct measurements independent of the DXA machines (Figure 3). The maximum coefficients of variation were previously reported to be 4.2%, 1.8% and 2.6%, respectively for NL, NSA and NW [54]. Covariates potentially influencing BMD and hip geometry were obtained at the time of DXA measurements along with an overall medical history. Details of these measurements have been reported previously [50]. These variables included age, sex, height, weight, and estrogen use/menopausal status (for women). Each woman was assigned to one of two estrogenic status groups: 1) premenopausal or postmenopausal on estrogen replacement therapy (estrogen-replete) or 2) postmenopausal not on estrogen (estrogen-deplete) where menopause was defined as having no menstrual period for at least one year.

#### Genotyping and exclusion of SNPs

Genotyping was conducted by the FHS SHARe (SNP Health Association Resource) project, for which 549,827 SNPs (Affymetrix 500K mapping array plus Affymetrix 50K gene center array) were genotyped in over 9,274 FHS subjects from over 900 families [55]. By estimation, we expected 80% genomic coverage (pair-wise genotype correlation *r* ^2^>0.8) of the HapMap Phase I+II common SNPs (minor allele frequency, MAF ≥0.05) for the Caucasian population [56]. We excluded 793 individuals with an average SNP call rate <0.97. We also excluded SNPs with call rate <0.95 (34,868 SNPs; 6.3%); Hardy-Weinberg equilibrium (HWE) test p-value <10^−6^ (8,531 SNPs; 1.6%); MAF <0.01 (66,829 SNPs; 12.2%); or unknown genomic annotation (6,089 SNPs; 1.1%). Ultimately, 433,510 SNPs were used in the genome-wide analyses.

#### Population substructure

Principal components analysis (PCA) was used to estimate population substructure in Framingham Study. We first applied PCA by EIGENSTRAT [57] to all available genotypic data to infer continuous axes of genetic variation (principal components, PCs) describing ancestral heterogeneity (top eigenvectors of a covariance matrix). Since the Framingham Study is family-based, the top 10 PCs were first built using a subset of 200 biologically unrelated subjects and projected to all study samples. The first two PCs showed gradients similar to those previously reported in individuals of European ancestry, such as northwest, southeast European and Ashkenazi Jewish [58]. Next, we assessed the association between top 10 PCs and each of the 5 phenotypes using regression models to examine if PCs were significantly associated with each phenotype with adjustment of age, sex, cohort, height and BMI. The top 4 PCs, PC1 to PC4 were all associated with FNBMD, LSBMD and NL at nominal p-values less than 0.05. However, the top 4 PCs together only accounted for 0.1∼0.7% of the variation in phenotypes. PCs were not significantly associated with NW and NSA. To account for potential population substructure in the SNP-phenotype association tests in Framingham Study, we adjusted PC1 to PC4 along with other covariates in the mixed effect regression models.

#### Statistical analysis

Sex- and cohort (Original and Offspring)-specific standardized residuals (mean = 0; SD = 1) of phenotypes were calculated using multivariate regression. For BMD phenotypes, the covariates adjusted in the regression models included PC1–PC4, age, age^2^ and estrogenic status (in women only). For hip geometry, the covariates included height, BMI, PC1–PC4, age, age^2^ and estrogenic status (in women only). Age^2^ was considered in the models to account for potential non-linear age effects. These residuals were used in association analyses described below. We performed both sex-specific and combined-sexes GWAS using linear mixed effects regression models (LME), with fixed SNP genotype effects, and random individual effects that correlate within pedigree according to kinship relationship [59]. The R package KINSHIP was used in the analyses. Although LME accounts for the within family correlation, like any population-based test for association, LME is sensitive to population admixture; therefore, PC adjusted residuals were used. Single SNP association tests were performed, using an additive genetic effect model that estimated the effect of one copy increment of the minor allele. To estimate how well the distribution was calibrated, for each phenotype, we estimated the genomic inflation factor (λ_GC_) based on the median chi-squared test of all study participants [60].

### Replication Stage (II)

Joint analysis for results from both discovery and replication stages almost always results in greater power than analyzing discovery and replication stages separately [61]. We selected SNPs with association test p-values less than 10^−3^ from Stage I discovery GWAS, and replicated them using meta-analysis by combining results from the Framingham Study and two independent population-based cohorts including the Rotterdam Study and the TwinsUK Study. Since both the Rotterdam and TwinsUK studies performed whole-genome genotyping using different platforms (Illumina platforms), SNP imputation was performed. Fixed effect meta-analyses were then used to estimate combined p-values.

#### Rotterdam Study

The Rotterdam Study is a prospective population-based cohort study of chronic disabling conditions in Dutch elderly individuals aged 55 years and over [62]. Microarray genotyping was performed in the whole original Rotterdam Study cohort using the Infinium II HumanHap550K Genotyping BeadChip version 3 (Illumina). The detail of genotyping procedures and quality control was reported elsewhere [27]. For population substructure, 102 individuals (>3 standard deviations) and 129 individuals (>97% probabilities) deviating from population mean of the IBS clustering [63] were excluded from association analysis. MACH [64], [65] was used to impute all autosomal SNPs from the HapMap I+II project. To account for the uncertainty of imputation, instead of using the “best guess” genotype for each individual, the additive dosage of the allele from 0 to 2, which is a weighted sum of the genotypes multiplied by their estimated probability, was used to perform association tests (MACH2QTL package). The ratio of the empirically observed dosage variance (from the imputed genotypes) to the expected (under binomial distribution) dosage variance (computed from the estimated minor allele frequency) was estimated for every SNP as a quality score for imputation. SNPs with the variance ratio <0.3 were excluded.

Age, gender and the distributions of phenotypes are shown in Table S1. Hip structural analysis measurements were done as described previously [66]. Sex-specific standardized residuals of phenotypes were calculated using general linear regression models adjusted for age, age^2^, height (for hip geometry only), and BMI (for hip geometry only). A linear regression model with additive genetic effect was used to estimate p-values for single SNP GWAS. The **λ_GC_** for each trait ranged from 0.98 to 1.06, suggesting that there was no major residual confounding by population stratification, systematic genotyping error, or little evidence of cryptic relatedness between individuals.

#### TwinsUK study

The TwinsUK cohort consists of approximately 10,000 monozygotic (MZ) and dizygotic (DZ) adult Caucasian twins aged 16 to 85 years recruited from the general population all over the United Kingdom [67]. This study was approved by the Research Ethics Committee of St. Thomas' Hospital, and written informed consent was obtained from each participant. BMD measurements (g/cm^2^) of the lumbar spine (L1–L4) and femoral neck were performed by DXA using a Hologic QDR 2000W densitometer (Hologic, Bedford, MA, USA). HSA software developed by Beck et al. [53] was used to measure hip geometry from the DXA scans as described in Framingham Study. The genotyping methods and quality control have been described previously [22]. In brief, 2,820 participants were genotyped by the Hap300Duo, Hap300 or Hap550 SNP Infinium assay (Illumina, San Diego, CA, USA). For potential population substructures, the STRUCTURE program was used to assess participants' ancestry genetic background [68]. After excluding 14 outliers (individuals) that lay outside the CEPH cluster from STRUCTURE analysis, the λ_GC_ for the distribution of test statistic of BMD and hip geometry ranged from 0.99 to 1.02, suggesting that there was no residual confounding by population stratification, nor any apparent systematic genotyping error, and little evidence of cryptic relatedness. IMPUTE [69] was used to impute all autosomal SNPs in the HapMap I+II project based on Map (release 22, build 26, CEU population) reference panel. The “best-guess” imputed genotypes were used in analyses. For each SNP, a confidence score was calculated as the average of the maximum posterior probabilities of the imputed genotypes. Individual genotypes with confidence score less than 0.9 were excluded.

2,734 women with both BMD and genotypes were in the final analyses (Table S1); however the sample size was smaller for HSA as these measurements have not been completed in all cohort members. Standardized residuals of phenotypes were calculated using general linear regression models adjusted for age, age^2^, height (for hip geometry only), and BMI (for hip geometry only). A score test implemented in MERLIN [70] was used to estimate p-values for single SNP analyses. An additive genetic effect model was tested.

#### Joint analysis using fixed effect meta-analysis model

We combined results from Framingham, Rotterdam and TwinsUK studies using inverse-variance fixed effect meta-analysis approaches to estimate combined p-values. The METAL program (http://www.sph.umich.edu/csg/abecasis/Metal/) was used. All association results were expressed relative to the forward strand of the reference genome based on HapMap (dbSNP126). The Cochran's *Q* heterogeneity test across studies was also estimated. Cochran's *Q* p-values less than 0.05 indicate large heterogeneity beyond chance. However, since only 2 or 3 cohorts were meta-analyzed, there was insufficient number of studies for the *Q* -statistics to be accurate calculated.

#### Multiple-testing

Recent GWAS have used different genome-wide significant thresholds in between 5×10^−7^ and 5×10^−8^ [71]–[76]. Several GWAS on multiple correlated traits estimated the genome-wide significant thresholds by FDR [73]–[76]. We performed gender-specific GWAS on 5 correlated traits (LS BMD, FN BMD, NSA, NL and NW). Since the pair-wise genetic correlation is 0.7 between LSBMD and FNBMD, Bonferroni correction for multiple testing is considered too conservative for correlated association tests. Therefore, we estimated genome-wide significant threshold by false discovery rate (FDR) [77]. A total of 4,336,025 association tests were performed in the discovery stage and in the meta-analysis replication stage. We then estimated the q-value (positive false-discovery rate) of each association test [78]. Based on the q-value and the number of significant tests (defined as an association test with q-value less than a particular q-value cutoff), we estimated the maximum number of false associations at each q-value cutoff. We set up the threshold for genome-wide significance of p-values as 4.3×10^−7^, and this threshold resulted in ≤1 expected false discovery of genome-wide significant association tests in our GWAS. The corresponding q-value is 0.011.

### Expression Profiling (Stage III): Human Tissues

We conducted expression quantitative trait locus (eQTL) analysis to evaluate whether the genome-wide significant SNPs for each locus also influence transcript levels of nearby genes as a *cis*-effect regulator (eSNP) in human primary osteoblasts, lymphocytes and liver tissue. In each locus, we selected nearby genes in which the genome-wide significant SNP was located within 500 Kb in the 5′ upstream of candidate genes with the assumption that SNPs are located in (or in LD with the variants located in) regulation elements of candidate genes. Expression experiments in primary osteoblasts, lymphocytes and liver tissues were conducted in three different study samples. For un-genotyped SNPs, imputed SNPs (MACH variance ratio >0.3) were used in the lymphocyte expression dataset and surrogate SNPs with LD r^2^≥0.5 were used in primary osteoblasts and liver tissue datasets.

#### Primary osteoblasts

A gene expression profile with 18,144 known genes (Illumina Human Ref8v2 BeadChips) and genome-wide genotyping of 561,303 SNPs (Illumina 550k Duo chips) were available in 95 human Caucasian primary osteoblast samples. Human trabecular bone from the shaft of proximal femora obtained from donors undergoing total hip replacement. Primary osteoblasts were derived from bone tissue. Tissue collection, RNA and DNA isolation, expression profiling, and DNA genotyping have been described in detail [79]. All gene expression levels were adjusted for sex and year of birth. We studied the *cis*-expression quantitative trait loci (cis-eQTL) of genome-wide significant SNPs or their proxy (eSNP) with selected transcripts within 500 kb of the SNP position. The linear regression model implemented in PLINK [63] was used to determine association between adjusted expression levels and genotypes.

#### Lymphocytes

Expression experiments in two different samples were performed. A gene expression profile with 20,599 genes (Affymetrix U133 Plus 2.0) and genome-wide genotyping of 408,273 SNPs (Illumina HumanHap300 Genotyping Beadchip) were available on 400 children from families recruited through a proband with asthma. The detailed study design was described elsewhere [9]. We also profiled expression levels using the Illumina Human 6 BeadChips on additional 550 children from the UK (recruited from families with atopic dermatitis probands). These individuals were genotyped using Illumina HumanHap300 Genotyping Beadchip. Inverse normal transformation was used to normalize the skewed distribution in both samples. MACH [64] was used to impute un-genotyped SNPs based on Phase II HapMap CEU panel. Association analysis was applied with FASTASSOC option implemented in MERLIN [80]. Only cis-effects within 500 Kb of the transcript were tested.

#### Liver tissue

A gene expression profile with 34,266 known genes (Agilent custom array) and genome-wide genotyping of 782,476 SNPs (Affymetrix 500K and Illumina 650Y SNP genotyping arrays) were available on 957 human Caucasian liver samples. Liver samples were either postmortem or surgical resections from organ donors. Tissue collection, RNA and DNA isolation, expression profiling, and DNA genotyping have been described previously [12]. All gene expression levels were adjusted for age, sex, race, and center. We studied the cis-eQTL of genome-wide significant SNP or its proxy (eSNP) with selected transcripts within 500 kb of the SNP position. The Kruskal-Wallis test was used to determine association between adjusted expression levels and genotypes.

### Expression Profiling (Stage III): Animal Models

#### Experiment 1: PTH stimulated gene expression profiling of mouse primary osteoblasts

Primary osteoblastic cultures were obtained from 2–3 day-old wild type C57BL/6J neonatal mice calvariae, half samples from males and half from females. Osteoblastic cell culture and PTH treatments were described elsewhere [81]. The 48-hour treatment cycle (incubation in medium with PTH for 6 hours, and incubation for the next 42 hours in medium without PTH) was repeated for 2 weeks. Cells were harvested and total RNA was isolated at day 14, after the last 6 hours of PTH exposure. Triplicate arrays were run for each condition/treatment with Affymetrix Mouse Genome 430A 2.0 arrays (approximately 14,000 genes per chip). Differences in gene expression levels between PTH and vehicle samples were evaluated. PTH-regulated genes were defined as follows: i) gene expression was detectable in all 3 PTH- and/or 3 vehicle-treated samples, ii) the average level of gene expression in PTH-treated samples was at least 1.5-fold higher or lower than in vehicle-treated samples, iii) gene expression levels differed by ≥1.5 fold between PTH and vehicle-treated samples in at least 7 out of 9 comparisons (each PTH-treated sample compared to vehicle-treated sample).

#### Experiment 2: Differential expression during osteoblast maturation

To determine whether the top associated genes were differentially expressed in maturing osteoblasts, we analyzed gene expression profiles of D3 murine embryonic stem cells that were undergoing directed differentiation toward the osteoblast lineage by treatment with vitamin D3, β-glycerophosphate and ascorbic acid. The gene expression dataset is publicly accessible via Gene Expression Omnibus, NCBI (GEO accession GSE3792). Gene expression patterns were generated using Affymetrix Mouse Genome 430A arrays at seven time points (Day 4, 5, 6, 8, 16, 25 and 30 post-induction). Triplicate arrays were run for each time point. The arrays were processed using the R AFFY package [82]. The robust multi-array average (RMA) algorithm was used for normalization [83]. ANOVA was used to identify genes whose expression differed across time points.

#### Experiment 3: Likelihood-based causality model selection

To identify causal relationships for the top associated genes discovered in our GWAS, we used the likelihood-based causality model selection (LCMS) algorithm by incorporating information of genotype, expression, and clinical traits together to construct regulatory networks. The description of the cross, genotyping and gene expression analysis have been described previously [12], [84]. The expression data is available via NCBI's Gene Expression Omnibus (GEO) database for adipose (GSE11065), liver (GSE11338) and muscle (GSE12795) tissues. The trabecular density measurement of the L_5_ vertebra from each F2 mouse was determined using a desktop μCT imaging system (μCT 40; Scanco Medical, Bassersdorf, Switzerland). The trabecular region was defined by contouring the inner section of the vertebral body with exclusion of the growth plate. Quantitative measurements of bone volume fraction (BV/TV), trabecular number (TbN), trabecular thickness (TbTh), trabecular separation (TbSp), mineral density of the bone volume fraction (DBV) and total femoral areal BMD (BMD) from mice were calculated using the Scanco software. μCT-derived trabecular bone data were evaluated against a hydroxyapatite standard in the same setting. LCMS procedure has been previously described [13], [31]. LCMS has been shown capable of recovering known causal relationships and we recently validated this approach by characterizing transgenic or knockout mouse models for 10 genes predicted causal for obesity by LCMS, seven of which significantly affected fat mass [85]. LCMS requires evidence of significant pleiotropy of eQTL/clinical trait QTL pairs using a likelihood modeling to test the fit of pleiotropy versus close linkage models. Three potential models were tested including: 1) Causal model: DNA variation affects a gene's expression which affects a clinical trait; 2) Reactive model: DNA variation affects a clinical trait which affects a gene's expression; and 3) Independent model: DNA variation independently affects both a gene's expression and clinical trait. The model with the lowest Bayesian Information Criteria (BIC) deemed the best fit. Reliability of each model call was determined by repeating LCMS on 1000 bootstrap samples. Candidate genes with at least 2 significant pleiotropy for eQTL and trait QTL pairs were considered to be causally related to differences in bone-related traits.

#### Experiment 4: Embryonic mouse *in vivo* gene expression atlas database

To determine where and when genes in the genome are expressed in the developing embryo *in vivo* [86], we ascertained the anatomic locations that genes were expressed during embryonic development in E10.5 and E14.5 wild type murine embryos from EURExpress database. Gene expression profiling on whole mounts and tissue sections of murine embryos were carried out by RNA *in-situ* hybridization with non-radioactive probes. Level and pattern of expression within each single organ or region are scored according to a standard scheme. Three different levels of expression were defined as (A) weak expression, (B) medium expression, and (C) strong expression. If no colored precipitate is seen, the gene expression is not detectable [87].

#### Prioritization of genome-wide suggestive candidate genes

To prioritize candidate genes from the list of suggestive genome-wide associated SNPs for further functional validation, we selected 109 suggestive candidate genes in which SNPs located within those regions were required to have all of the following criteria: (1) meta-analysis p-values of association test in the range of 4.3×10^−7^<p≤5×10^−5^, (2) p-values from the discovery stage <10^−3^, (3) p-values ≤0.05 from the replication stage and (4) the same direction of effect from both discovery and replication stages. We then prioritized these candidate genes based on results from either (1) two significant results supported by three expression signature profiles including PTH regulated genes (+++) from experiment 1, differentiated expression during osteoblast maturation (p<4.59×10^−4^, Bonferroni correction for 109 tests) from experiment 2 and at least moderate expression in skeletal sites of mouse embryos from experiment 4; or (2) candidate genes with at least 2 pairs of significantly pleiotropic QTL/eQTL effects from LCMS modeling (experiment 3).

### Bioinformatic Approaches (Stage IV)

#### Gene-set enrichment tests on functional similarity

To explore functional similarity of our prioritized associated genes, we performed a gene-set enrichment test to examine the probability of our candidate genes clustering in particular biological/functional pathways as defined by the Gene Ontology (GO) project [88]. The GO Consortium provides controlled vocabularies, which model “Biological Process”, “Molecular Function” and “Cellular Component” that are structured into directed acyclic graphs based on published literature and databases. Gene products may be annotated to one or more GO nodes. To determine whether any GO terms annotate a specified list of genes at a frequency greater than that would be expected by chance, a p-value was calculated using the hyper-geometric distribution [89]. To correct for multiple testing, false discovery rate (FDR) was estimated [77].

#### Gene-set enrichment tests on expression abundance in human tissues

It has been well accepted that the content of the expressed sequence tag (EST) pool for a given tissue type reflects the composition of original mRNA samples used for creation of the complementary DNA library [90]. We estimated gene expression abundance for our top associated genes in an EST cDNA library (48 types of human normal tissues and organs) from the Cancer Genome Anatomy Project, National Cancer Institute (http://cgap.nci.nih.gov/Tissues). We estimated the expected expression levels and performed hyper-geometric tests to evaluate over- or under-representation of individual genes in selected tissues, including bone, liver, muscle and adipose tissue.

## Supporting Information

Figure S1 Observed and expected expression levels (number of EST sequences) in human bone and liver tissues from cDNA Library. * 10^−10^≤p<0.0017; ** 10^−20^≤p<10^−10^; *** p<10^−20^.
(0.46 MB TIF)

Figure S2 Observed and expected expression levels (number of EST sequences) in human muscle and adipose tissues from cDNA Library. * 10^−10^≤p<0.0017; ** 10^−20^≤p<10^−10^; *** p<10^−20^.
(0.42 MB TIF)

Figure S3 Relative transcript levels (standardized residuals) in human liver tissue for top associated candidate genes (from meta-analysis) by genotype of top associated SNPs (or proxy) in Table 2.
(0.19 MB TIF)

Table S1 Descriptive characteristics of study participants by Cohorts.
(0.02 MB XLS)

Table S2 SNPs with association test p-value <1.0E-06 in the Framingham Study.
(0.02 MB XLS)

Table S3 Molecular and functional characteristics of the selected candidate genes in each region reported in Table 2.
(0.02 MB XLS)

Table S4 Genes were predicted to be causally linked with bone phenotypes using the Likelihood-based Causality Model (LCMS).
(0.02 MB XLS)

Table S5 Molecular and functional characteristics of the selected candidate genes in each region reported in Table 4.
(0.03 MB XLS)
