# Supplementary figures and images for: An Integration of Genome-Wide Association Study and Gene Expression Profiling to Prioritize the Discovery of Novel Susceptibility Loci for Osteoporosis-Related Traits
Source: PLoS Genet. 2010 Jun 10;6(6):e1000977. doi: 10.1371/journal.pgen.1000977 (PMC2883588; doi:10.1371/journal.pgen.1000977)

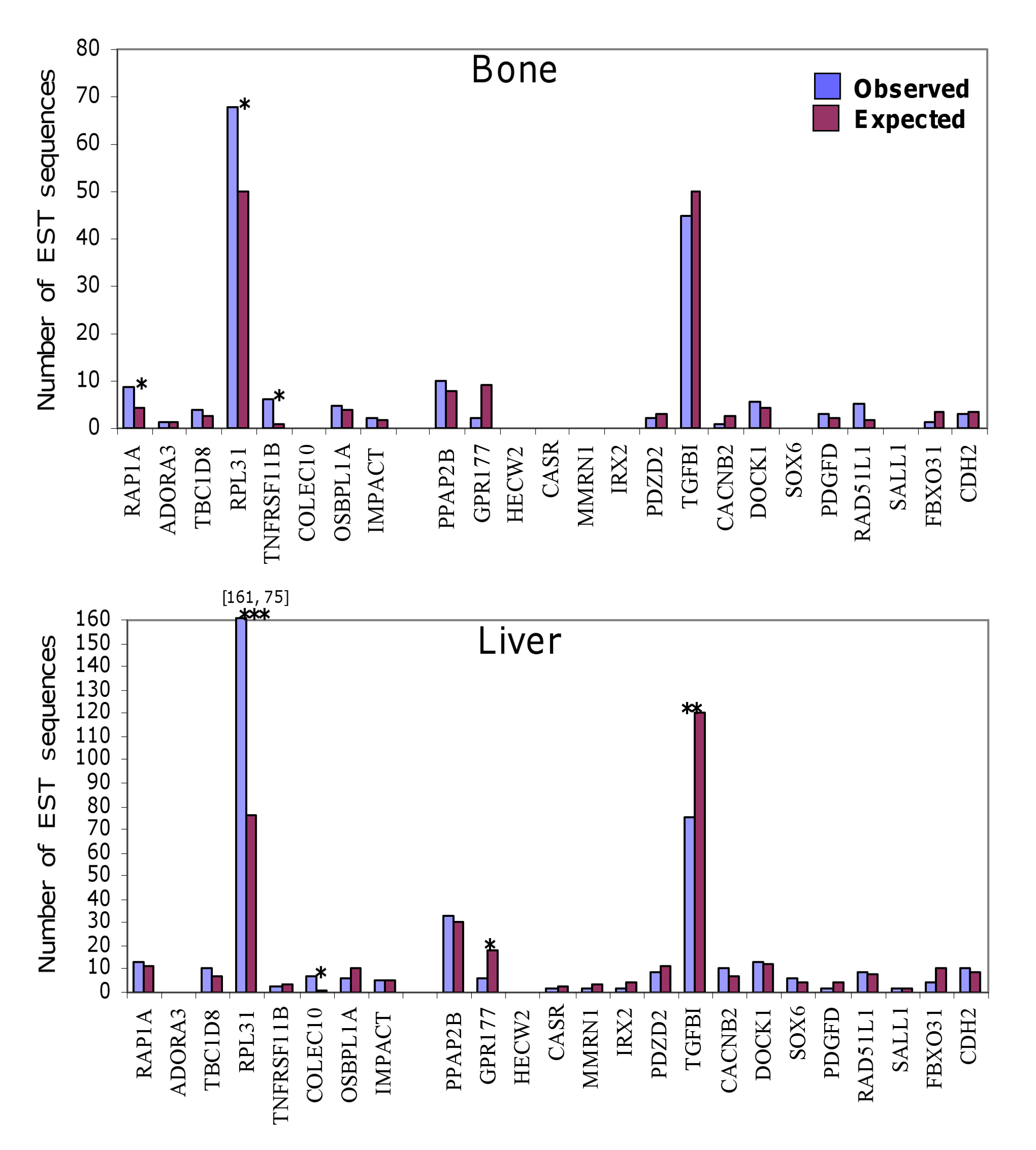

Supplement: Figure S1 — Observed and expected expression levels (number of EST sequences) in human bone and liver tissues from cDNA Library. * 10−10≤p<0.0017; ** 10−20≤p<10−10; *** p<10−20. (0.46 MB TIF) [file pgen.1000977.s001.tif]

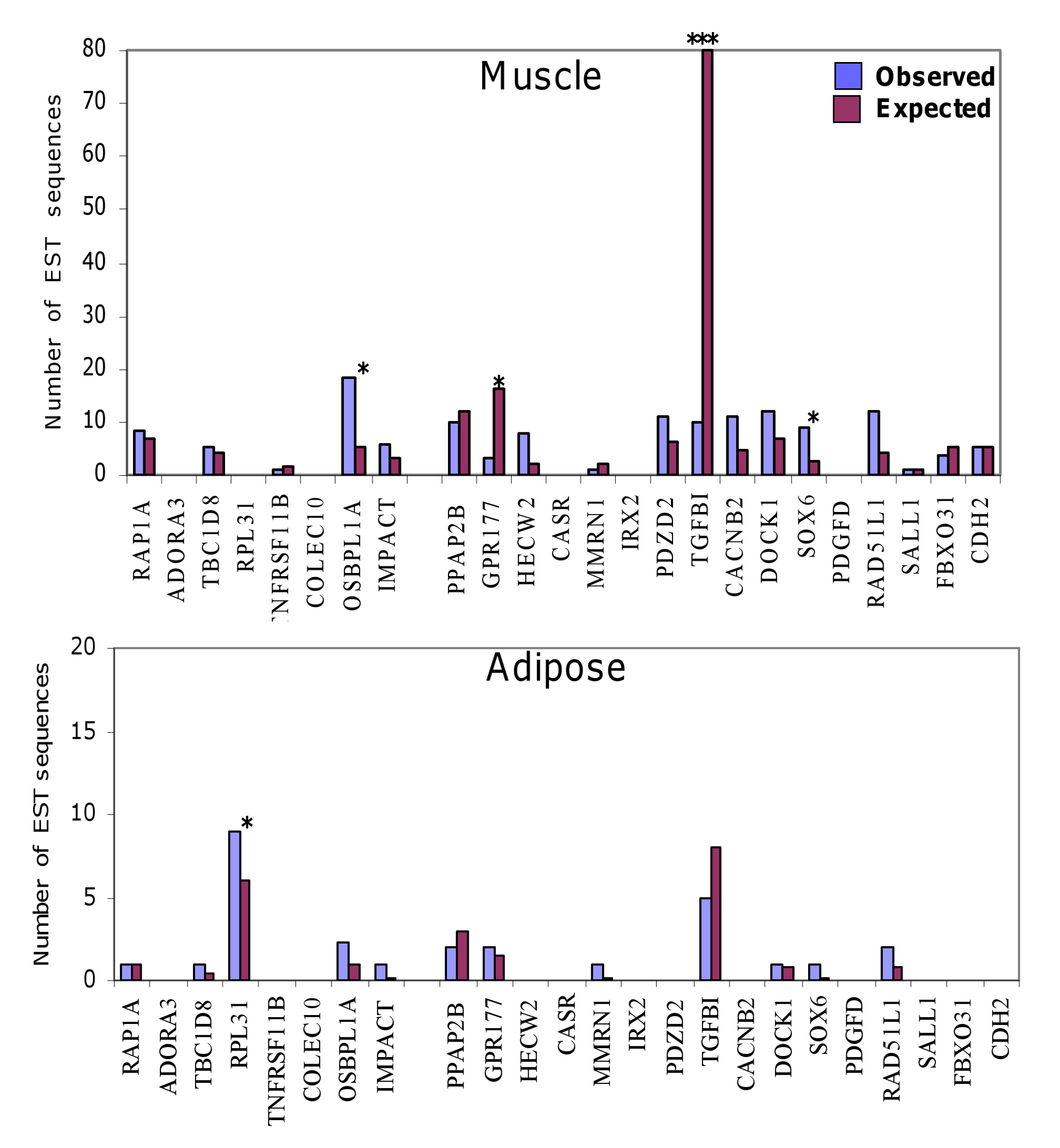

Supplement: Figure S2 — Observed and expected expression levels (number of EST sequences) in human muscle and adipose tissues from cDNA Library. * 10−10≤p<0.0017; ** 10−20≤p<10−10; *** p<10−20. (0.42 MB TIF) [file pgen.1000977.s002.tif]

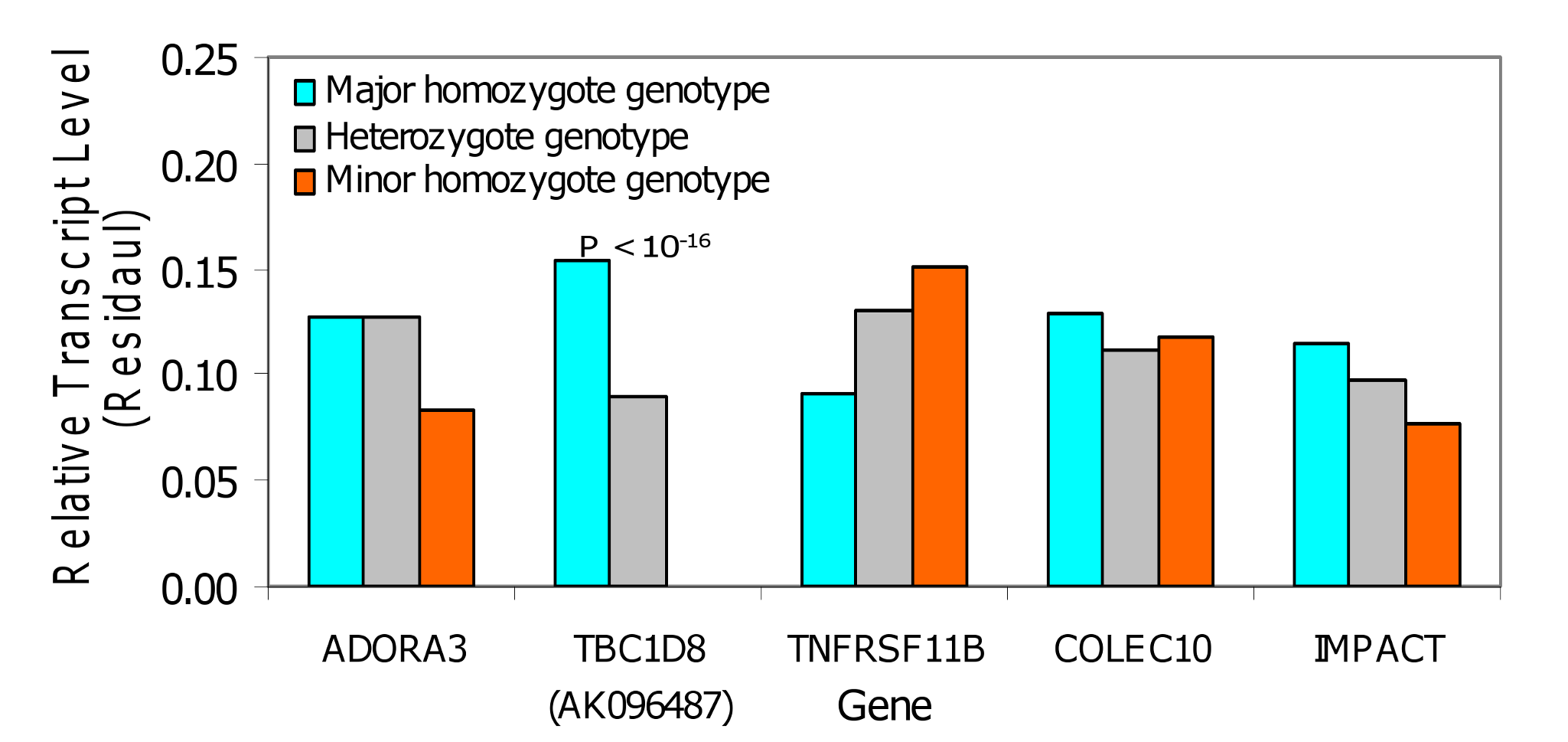

Supplement: Figure S3 — Relative transcript levels (standardized residuals) in human liver tissue for top associated candidate genes (from meta-analysis) by genotype of top associated SNPs (or proxy) in Table 2. (0.19 MB TIF) [file pgen.1000977.s003.tif]
